# Patient with sick sinus syndrome and implanted dual-chamber pacemaker with reduced P-wave duration following low interatrial septal pacing: Case report

**DOI:** 10.1097/MD.0000000000027076

**Published:** 2021-09-03

**Authors:** Jakub Mercik, Aleksandra Gajek-Marecka, Jacek Marcin Zawadzki, Agnieszka Sławuta, Jacek Gajek

**Affiliations:** a Department of Emergency Medicine, Wroclaw Medical University, Wroclaw, Poland; b Klodzko County Hospital, Klodzko, Poland; c Department of Anesthesia, Critical Care and Emergency Medicine, Collegium Medicum of University in Zielona Góra, Zielona Góra, Poland; d Department of Internal and Occupational Diseases, Hypertension and Clinical Oncology, Wroclaw Medical University, Wroclaw, Poland; e Department of Emergency Medical Service, Wroclaw Medical University, Wroclaw, Poland.

**Keywords:** coronary sinus, interatrial septum, pacing, sick sinus syndrome

## Abstract

**Introduction:**

A dual-chamber pacemaker (DDD/R) for a sinus node disease is sometimes referred to as a physiological pacemaker as it maintains atrioventricular synchrony, however several clinical trials have proved its inferiority to a nonphysiological single-chamber ventricular back-up pacing.

**Patient concerns:**

A subject of the study is a 74-year-old woman with a sick sinus syndrome (SSS) and a previously implanted physiological DDD/R pacemaker. The SSS was diagnosed because of patient's very slow sinus rhythm of about 36 bpm, and due to several episodes of dizziness. After the DDD/R implantation the percentage of atrial pacing approached 100%, with almost none ventricular pacing.

**Diagnoses:**

Sick sinus syndrome, complete Bachmann's bundle block, atrial fibrillation, atrial flutter.

**Interventions:**

The patient was previously implanted with a physiological DDD/R pacemaker. Several years after the implantation, the atrial fibrillation was diagnosed and the pulmonary vein isolation was then performed by cryoablation. During the follow-up after pulmonary vein isolation, the improvement of mitral filling parameters was assessed using echocardiography. Shortly thereafter the patient developed the persistent paroxysm of a typical atrial flutter which was successfully terminated using a radiofrequency ablation. No recurrence thereof has been observed ever since (24 months).

**Outcomes:**

The atrial electrode of the pacing system was implanted within the low interatrial septal region that resulted in a reduced P-wave duration compared to native sinus rhythm P-waves. The said morphology was deformed because of the complete Bachmann bundle block. That approach, despite a nonphysiological direction of an atrial activation, yielded relatively short P-waves (paced P-wave: 179 ms vs intrinsic sinus P-wave: 237 ms). It also contributed to a significantly shorter PR interval (paced PR: 204 ms vs sinus rhythm PR: 254 ms).

**Conclusions:**

The authors took into consideration different aspects of alternative right atrial pacing sites. This report has shown that in some patients with a sinus node disease, low interatrial septal pacing can reduce the P-wave duration but does not prevent from the development of atrial arrhythmias.

## 1 Introduction

A dual-chamber pacemaker can be used to manage various types of symptomatic bradycardia. This device makes it possible to sense the activity of both the atrium and the ventricle, and to pace both of them, thus helping to maintain atrioventricular synchrony. This kind of pacing is referred to as a physiological pacing.^[1]^ Despite the physiological nature of dual-chamber pacing, the implantation of dual-chamber pacemakers did not result in key outcome results in several studies regarding stroke and survival but it slightly improved the risk of atrial fibrillation (AF), signs of heart failure, and the quality of life.^[2–4]^

A proper recognition of a sinus node disease whose symptomatic form is referred to as a sick sinus syndrome is of high importance. This clinical condition, if not properly treated, might contribute to the development of paroxysmal or chronic AF and its subsequent complications, to the heart failure and to other disabilities.^[5]^ The atrial pacing that is ensured by the artificial pacemaker can influence the atrial activation, interatrial conduction, and finally the P-wave morphology.^[6]^ The standard location of an atrial electrode placement—the right atrial appendage—prolongs the interatrial conduction and the P-wave duration. Furthermore, the interatrial conduction can be impaired by an incomplete and a complete Bachmann bundle block.^[7]^ The interatrial conduction disorders are relatively frequent in particular in older patients with AF and contribute to the final permanent maintenance of the arrhythmia.^[8]^

The Bachmann bundle pacing or a high interatrial septal pacing produces a much shorter P-wave as the impulse activates both atria simultaneously.^[9]^ It is still unknown whether the Bachmann bundle pacing could correct the complete Bachmann bundle block.

The authors aimed at drawing considerations with regards to various strengths and shortcomings of a low atrial septum/ coronary sinus ostium pacing in our patient.

## 2 Case description

**Figure 1:**
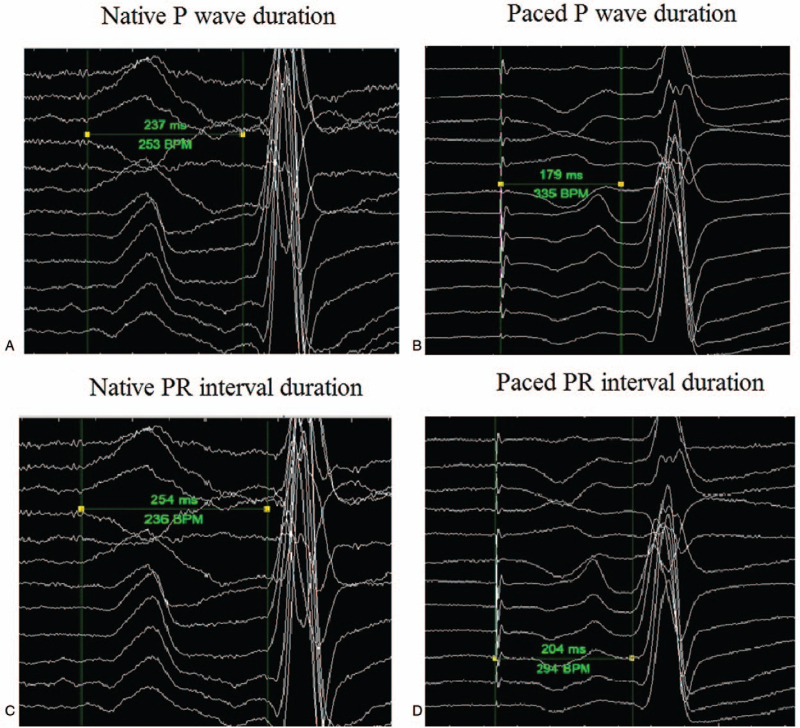
P wave\PR interval characteristics.

We present a 74-year-old female patient with a previously implanted dual-chamber pacemaker for a sinus node disease. The SSS was diagnosed according to the guidelines recommendations, because of a patient's very slow rhythm of about 36 bpm, and due to a few episodes of dizziness.^[10,11]^ After the DDD/R implantation the percentage of atrial pacing approached 100%, with almost none ventricular pacing. Due to the complete Bachmann bundle block, the atrial electrode was actively fixed in the lower part of the interatrial septum, in the region of the coronary sinus ostium, which was in concordance with the literature reports.^[12]^ That approach, despite a nonphysiological direction of an atrial activation, yielded relatively short P-waves (paced P-wave: 179 ms vs intrinsic sinus P-wave: 237 ms). It also contributed to a significantly shorter PR interval (paced PR: 204 ms vs sinus rhythm PR: 254 ms). The described ECG features are presented in Figure 1.

**Figure 2:**
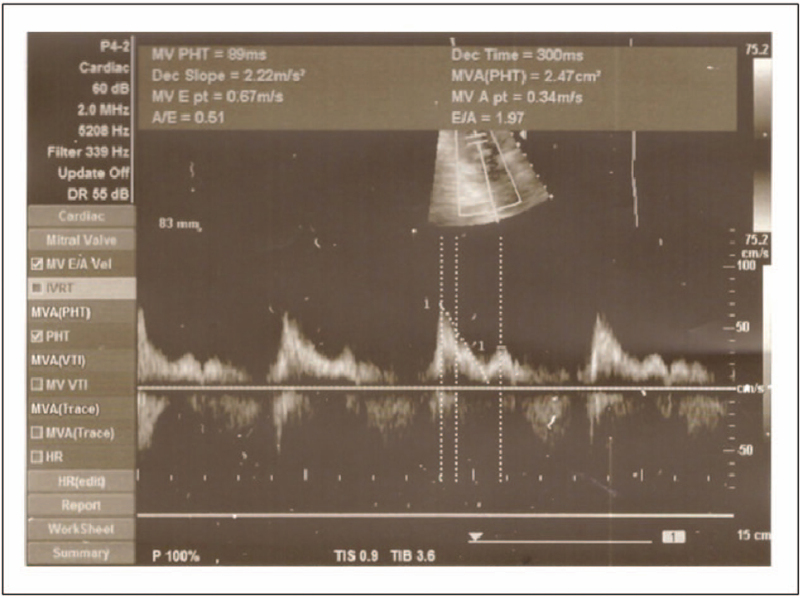
Mitral flow parameters.

The patient presented twice in our facility, each time with a different arrhythmia. In September 2018 the AF was diagnosed, and the pulmonary veins isolation was performed simultaneously with a direct current cardioversion to terminate the arrhythmia. In a 4-month follow-up after the pulmonary veins isolation, the mitral filling parameters improved (E/A = 1.97; E wave = 0.67 m/s; A wave 0.34 m/s) which could indicate the incomplete recovery of the left atrial mechanical function, but more probably it reflected the nonphysiological direction of an atrial mechanical contraction. The mitral filling assessment was depicted in Figure 2.

In April 2019 the persistent paroxysm of a typical atrial flutter (AFL) was diagnosed, and radiofrequency ablation successfully terminated it without any recurrences. Now, after the 24-month follow-up, the patient does not complain about any palpitations or signs and symptoms of a heart failure. The appropriate function of the pacemaker is monitored on a regular basis.

## 3 Discussion

The right and the left atria are activated almost simultaneously (within 50–80 ms) during the sinus rhythm. The places of electrical connections between the atria are the Bachmann bundle, the Koch triangle region, the fossa ovalis, and the coronary sinus. The site of the artificial pacing that is the closest to the physiological conduction between the atria is the Bachmann bundle area. Jurkko et al in their study using magnetocardiography confirm that in most cases of atrial stimulation, the activation of the left atrium occurs via the Bachmann bundle as a lonely path or concomitantly with other ways.^[13]^ In the presented case, the total Bachmann bundle block did not allow to place the atrial electrode physiologically. For this reason the operators had to use a different approach.^[14]^ The low interatrial septum pacing was used in some dedicated trails which gave inconsistent results. In the SAFE study by Lau et al, in 385 patients with a paroxysmal AF and SSS requiring a pacemaker implantation, an alternative atrial pacing site in the right atrial septum and a continuous atrial overdrive pacing did not prevent from the development of a persistent AF (11). Contrary to the aforementioned results, Verlato et al in the EPASS study in the group of 102 patients proved some benefits in preventing progression to a persistent or a permanent AF.^[10]^

If the Bachmann bundle pacing is impossible due to a damage to the bundle area, it seems reasonable to position the electrode in the lower part of the interatrial septum/coronary sinus ostium. The duration of the P-wave in the study of de Vogt et al was longer in the right atrial appendage pacing compared to the lower part of the interatrial septum/coronary sinus ostium (115 ± 19 vs 80 ± 14 ms).^[15]^ Padeletti et al examined 46 patients with a paroxysmal AF who were randomized for either the right atrial appendage or the lower part of the interatrial septum/coronary sinus ostium pacing. The superiority of the last location in reducing AF relapses over 3 months was observed.^[16]^ Similar results were obtained by the already mentioned Verlato et al who studied the effect of the electrode placement in preventing a persistent AF in patients with a sinus node disease. The subjects were assigned to respective study groups according the interatrial conduction time. In only 2 of 29 patients, the lower part of the interatrial septum/coronary sinus ostium pacing resulted in a persistent AF whereas the same but with the right atrial appendage pacing was observed in 9 of 36 patients.^[10]^

Patients may also benefit from the pacing of the lower part of the interatrial septum in comparison to the right atrial appendage pacing in terms of atrial hemodynamics. In the study of Wang et al, the authors measured peak atrial velocities using the tissue Doppler imaging. During atrial pacing from the lower part of the interatrial septum/coronary sinus ostium, the left atrial ejection fraction was better compared to right atrial appendage pacing (52% ± 16% vs 39% ± 14%). The local velocity measured on the free right atrium wall (14.3% ± 3.1% vs 10.3 ± 4.4 cm/s), the septum (7.5 ± 2.1 vs 5.2 ± 1.7 cm/s) and on the left lateral wall (8.6 ± 2.4 vs. 6.3 ± 3.0 cm/s) was also in favor of the interatrial septum pacing. The authors of the study concluded that the pacing of these places improves a global and a regional mechanical function of the atria and a synchronized contraction compared to the pacing from the right atrial appendage.^[17]^ However, the study of Kugacka-Dąbrowska et al in an acute echocardiographic examination performed in 15 healthy subjects and in 25 patients with a sinus node dysfunction and a recurrent AF indicated the important hemodynamic differences during the multisite atrial pacing. The pacing of the lower part of the interatrial septum/coronary sinus ostium resulted in the right atrial filling impairment, it shortened the mechanical atrioventricular delay in the right heart and diminished the right ventricular inflow. The Bachmann bundle pacing provided the best atrial contraction synchrony. The authors concluded that a single-site lower part of the interatrial septum/coronary sinus ostium pacing induced the echocardiographic pacemaker syndrome in the right heart.^[18]^

The approach that we are presenting in this procedure involves both positive and negative effects of the pacing of the lower part of the interatrial septum/coronary sinus ostium in our patient, and as such they need to be taken into consideration.

The atrial pacing makes it possible to maintain the appropriate heart rate and the cardiac output in patients with a profound sinus bradycardia. This offers also the possibility of an antiarrhythmic treatment in patients with AF, something otherwise not possible due to “bradycardiogenic" effects of nearly all antiarrhythmic medications. The additional result of a constant pacing is also some antiarrhythmic effect by the heart rate stabilization.^[19]^

The negative consequences of the low atrial septum/ coronary sinus pacing are related mainly to the altered, nonphysiological sequence of an atrial activation which in turn alter the mechanical coupling, atrial emptying, and ventricular diastolic filling.^[18]^

In the presented case, the location of the pacing electrode in the lower part of the interatrial septum/coronary sinus ostium region resulted in a significant shortening of the P-wave and of the atrioventricular conduction in the form of a shorter PR interval. Nonetheless, the resulting P-wave duration was not 120 ms. It is difficult to assess if this approach had been of any additional value in this particular patient (except for the anti-bradycardia pacing) because the AF and the AFL occurred in the follow-up period and were successfully treated by the ablation. The literature data prove that the lower part of the interatrial septum/coronary sinus ostium pacing is superior over the right atrial appendage pacing. Any specific impact of this pacing site on the right atrium mechanics and on the AFL facilitation is yet unknown but it might be considered with regards to the echocardiographic data.^[20]^

## 4 Conclusions

1. The lower part of the interatrial septum/coronary sinus ostium pacing results in a shortening of the P-wave duration as well as of the PR interval.
2. It is difficult to assess whether any specific group of patients (for instance those who present with the complete Bachmann's bundle block) might find this approach to be beneficial.
3. This report has shown that for some patients with the sinus node disease, the low interatrial septal pacing can reduce the duration of the P-wave; however, it does not prevent from atrial arrhythmias.

## Author contributions

All authors collected and recorded the original data, analyzed the case and wrote the manuscript. JG guided all work.

**Conceptualization:** Jakub Mercik, Aleksandra Gajek-Marecka, Jacek Marcin Zawadzki, Agnieszka Sławuta, Jacek Gajek.

**Data curation:** Jakub Mercik, Aleksandra Gajek-Marecka, Jacek Marcin Zawadzki, Agnieszka Sławuta, Jacek Gajek.

**Formal analysis:** Jakub Mercik, Aleksandra Gajek-Marecka, Jacek Marcin Zawadzki, Agnieszka Sławuta, Jacek Gajek.

**Funding acquisition:** Jakub Mercik, Aleksandra Gajek-Marecka, Jacek Marcin Zawadzki, Agnieszka Sławuta, Jacek Gajek.

**Investigation:** Jakub Mercik, Aleksandra Gajek-Marecka, Jacek Marcin Zawadzki, Agnieszka Sławuta, Jacek Gajek.

**Methodology:** Jakub Mercik, Aleksandra Gajek-Marecka, Jacek Marcin Zawadzki, Agnieszka Sławuta, Jacek Gajek.

**Project administration:** Jakub Mercik, Aleksandra Gajek-Marecka, Jacek Marcin Zawadzki, Agnieszka Sławuta, Jacek Gajek.

**Resources:** Jakub Mercik, Aleksandra Gajek-Marecka, Jacek Marcin Zawadzki, Agnieszka Sławuta, Jacek Gajek.

**Software:** Jakub Mercik, Aleksandra Gajek-Marecka, Jacek Marcin Zawadzki, Agnieszka Sławuta, Jacek Gajek.

**Supervision:** Jakub Mercik, Aleksandra Gajek-Marecka, Jacek Marcin Zawadzki, Agnieszka Sławuta, Jacek Gajek.

**Validation:** Jakub Mercik, Aleksandra Gajek-Marecka, Jacek Marcin Zawadzki, Agnieszka Sławuta, Jacek Gajek.

**Visualization:** Jakub Mercik, Aleksandra Gajek-Marecka, Jacek Marcin Zawadzki, Agnieszka Sławuta, Jacek Gajek.

**Writing – original draft:** Jakub Mercik, Aleksandra Gajek-Marecka, Jacek Marcin Zawadzki, Agnieszka Sławuta, Jacek Gajek.

**Writing – review & editing:** Jakub Mercik, Aleksandra Gajek-Marecka, Jacek Marcin Zawadzki, Agnieszka Sławuta, Jacek Gajek.
